# Retraction: MIR600HG suppresses metastasis and enhances oxaliplatin chemosensitivity by targeting ALDH1A3 in colorectal cancer

**DOI:** 10.1042/BSR-2020-0390_RET

**Published:** 2024-09-10

**Keywords:** ALDH1A3, Chemosensitivity, colorectal cancer, Metastasis, MIR600HG

This article is being retracted from *Bioscience Reports* at the request of the Editor-in-Chief and the Editorial Board following receipt of a notification from a reader, alerting the Editorial Board to multiple images in this paper that seem to appear in a subsequent paper by different authors. Specifically, Figure 2F siMIR600HG panel, Figure 6D NC panel, Figure 6D MIR600HG panel, and Figure 6D MIR600HG+ALDH1A3 panel seem to appear in a paper by Zhong et al, 2020 (DOI: 10.1042/BSR20200367) [retracted].

Upon review of the case, the Editorial Office identified further links to the paper by Zhong et al, 2020 (DOI: 10.1042/BSR20200367) [retracted], and also believe that the peer review process was likely compromised for this article. The authors were contacted regarding the image concerns. The authors stated the original data were damaged and provided a replacement Figures 2F and Figure 6D. However, given the image duplication and peer review concerns, the Editorial Board stands by the decision to retract. The authors disagree with the retraction.

